# Temperature Compensation Method for MEMS Ring Gyroscope Based on PSO-TVFEMD-SE-TFPF and FTTA-LSTM

**DOI:** 10.3390/mi16050507

**Published:** 2025-04-26

**Authors:** Hongqiao Huang, Wen Ye, Li Liu, Wenjing Wang, Yan Wang, Huiliang Cao

**Affiliations:** 1 Key Laboratory of Instrumentation Science & Dynamic Measurement, Ministry of Education, North University of China, Taiyuan 030051, China; s202206084@st.nuc.edu.cn; 2 National Institute of Metrology, China, 18 North Third Ring East Road, Chaoyang District, Beijing 100029, China; yewen@nim.ac.cn; 3 Mechanized Infantry Reconnaissance Department, The Army Infantry Academy of PLA, Shijiazhuang 050083, China; liuli11280713@163.com; 4 School of Microelectronics, University of Science and Technology of China, Hefei 230022, China; w520480@126.com; 5 Automobile NCO School, Army Military Transportation University, No. 1155 Yanshan Road, Yuhui District, Bengbu 233010, China

**Keywords:** MEMS ring gyroscope, temperature compensation, noise

## Abstract

This study proposes a novel parallel denoising and temperature compensation fusion algorithm for MEMS ring gyroscopes. First, the particle swarm optimization (PSO) algorithm is used to optimize the time-varying filter-based empirical mode decomposition (TVFEMD), obtaining optimal decomposition parameters. Then, TVFEMD decomposes the gyroscope output signal into a series of product function (PF) signals and a residual signal. Next, sample entropy (SE) is employed to classify the decomposed signals into three categories: noise segment, mixed segment, and feature segment. According to the parallel model structure, the noise segment is directly discarded. Meanwhile, time–frequency peak filtering (TFPF) is applied to denoise the mixed segment, while the feature segment undergoes compensation. For compensation, the football team training algorithm (FTTA) is used to optimize the parameters of the long short-term memory (LSTM) neural network, forming a novel FTTA-LSTM architecture. Both simulations and experimental results validate the effectiveness of the proposed algorithm. After processing the MEMS gyroscope output signal using the PSO-TVFEMD-SE-TFPF denoising algorithm and the FTTA-LSTM temperature drift compensation model, the angular random walk (ARW) of the MEMS gyroscope is reduced to 0.02°/√h, while the bias instability (BI) decreases to 2.23°/h. Compared to the original signal, ARW and BI are reduced by 99.43% and 97.69%, respectively. The proposed fusion-based temperature compensation method significantly enhances the temperature stability and noise performance of the gyroscope.

## 1. Introduction

The rapid advancement of micro-electro-mechanical systems (MEMS) technology has significantly promoted the progress of inertial sensing devices, with MEMS gyroscopes becoming essential components in high-precision applications ranging from aerospace to industrial automation [1,2]. Their compact size, low power consumption, and seamless integration capability make them indispensable in modern systems [3]. However, applications such as aerospace and weaponry impose more stringent requirements on MEMS gyroscope performance, particularly concerning noise performance and bias stability [4]. Manufacturing processes, hardware design, and inherent sensor defects lead to notable deviations between the gyroscope output signal and its ideal output. These deviations considerably degrade the measurement accuracy of MEMS gyroscopes [5]. Currently, the primary sources of errors include both internal and external system noise and output drift caused by temperature fluctuations. Therefore, temperature is one of the crucial environmental factors affecting MEMS gyroscope performance [6].

Temperature variations can cause changes in the material properties of MEMS gyroscope structures, such as Young’s modulus, indirectly affecting parameters like the gyroscope’s quality factor [7]. Additionally, gyroscope drive and detection circuits are highly sensitive to temperature, primarily due to orthogonal errors resulting from stiffness mismatches [8]. Without effective temperature compensation, MEMS gyroscopes cannot meet the requirements of high-precision applications. Therefore, extensive research has been dedicated to developing temperature error compensation methods for MEMS gyroscopes [9].

Existing temperature error compensation methods can be categorized into two types: hardware compensation based on structural and circuit modifications and software-based data-driven approaches. Hardware-based compensation methods include optimizing gyroscope sensing structures and selecting materials by analyzing critical parameters, such as the resonance frequency, to reduce the impact of temperature on gyroscope performance. Nevertheless, significant structural design improvements often require extensive research and experimentation and may increase complexity and cost [10,11]. Some studies employ control circuits for on-chip temperature compensation through automatic calibration of frequency mismatches, achieving substantial suppression of temperature drift [12,13]. However, these hardware-focused methods generally cannot address drifts and noise introduced by control circuits themselves and typically entail high cost, difficulty in implementation, and prolonged development cycles.

Existing temperature error compensation methods based on algorithms are classified into serial and parallel frameworks. Serial approaches typically perform denoising prior to drift compensation, potentially losing temperature-related signal characteristics during initial filtering. In contrast, parallel structures decouple noise and temperature drift through adaptive signal decomposition and classification [14]. Reference [15] employs the second-order autoregressive moving average (ARMA) method to establish a temperature error model for the gyroscope. However, this approach demonstrates limited effectiveness in suppressing high-frequency noise in the gyroscope’s output signal. References [16,17] model temperature errors using neural networks (RBFs), variational mode decomposition (VMD), and empirical mode decomposition (EMD). However, these algorithms exhibit limited decomposition performance and poor network prediction accuracy. For instance, variational mode decomposition (VMD) requires careful parameter selection, including the number of modes and balance parameters, which significantly impact decomposition quality [18]. Meanwhile, empirical mode decomposition (EMD) is prone to mode-mixing issues [19]. Traditional radial basis function (RBF) networks struggle to handle the complex nonlinear characteristics of temperature-related errors in MEMS gyroscopes, especially when these relationships vary significantly under different operating conditions [20].

The latest methods focus on proposing different temperature error compensation schemes for MEMS gyroscopes based on signal decomposition and artificial intelligence fusion. Chen [21] proposed a temperature drift suppression method combining mode reversal and multivariate regression. The mode reversal eliminates the zero-bias drift caused by the damping axis offset and, in combination with real-time multivariate regression, compensates for residual drift under rapid temperature changes, resulting in a 61-fold improvement in bias stability and a 46-fold reduction in bias variation rate. Wang [22] developed an improved VMD-ELM algorithm that integrates CNN-LSTM and PSO-SVM technologies. They utilized VMD to decompose the gyroscope output signal into high, medium, and low-frequency components, which were then modeled and optimized through CNN-LSTM and PSO-SVM, respectively. The signals were reconstructed using ELM, reducing quantization noise (Q) and bias instability (B) to 0.85% and 2.16% of their original values. Guo [23] proposed a compensation method for MEMS accelerometers combining DLSTM-RNN and an improved sparrow search algorithm (ISSA). Through piecewise linear approximation and real-time compensation model optimization for temperature drift, the method improved bias instability, rate random walk, and rate slope average by 96.68%. Wang [24] developed a zero-bias temperature compensation fusion algorithm based on RIME-VMD-PSO-DBN, which reduced zero-bias instability from 49.32°/h to 0.98°/h.

To address these issues, this study proposes a novel parallel denoising and temperature compensation fusion algorithm based on PSO-TVFEMD-SE-TFPF and FTTA-LSTM models. Firstly, the particle swarm optimization algorithm (PSO) optimizes the time-varying filter-based empirical mode decomposition (TVFEMD) algorithm, achieving optimal decomposition parameters. Subsequently, the optimized PSO-TVFEMD algorithm decomposes the gyroscope output signal into product function (PF) signals and residual signals. Next, sample entropy (SE) classifies these decomposed signals into noise segments, mixed segments, and characteristic segments. According to the parallel model requirements, noise segments are directly discarded, while mixed segments are denoised using time–frequency peak filtering (TFPF), and characteristic segments undergo compensation. The football team training algorithm (FTTA) is utilized to optimize the parameters of the LSTM neural network (LSTM), proposing a novel FTTA-LSTM structure. Experimental results demonstrate that the proposed PSO-TVFEMD-SE-TFPF denoising algorithm and FTTA-LSTM temperature compensation model significantly improve the bias instability (BI) by 97.69% and angular random walk (ARW) by 99.43%, confirming excellent performance in temperature compensation and further enhancing the MEMS ring gyroscope’s temperature stability.

The organization of this paper is as follows: Section 2 introduces the hardware structure of the MEMS ring gyroscope. Section 3 presents the detailed composition of the PSO-TVFEMD-SE-TFPF and FTTA-LSTM models, along with the fusion algorithm steps. Section 4 showcases the experimental results and provides a comparative analysis of different algorithms. Section 5 concludes the study.

## 2. Introduction to MEMS Ring Gyroscope

**Figure 1:**
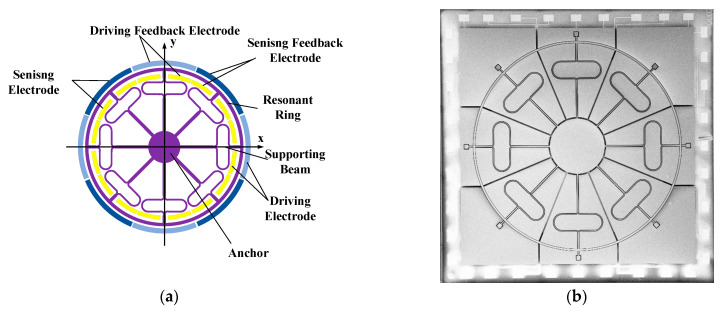
MEMS ring gyroscope structure: (**a**) schematic diagram; (**b**) SEM image.

The experimental signals utilized in this study were collected from a MEMS ring gyroscope independently developed by our institution [25], as shown in Figure 1. This gyroscope was fabricated using (111) single-crystal silicon wafers and borosilicate glass wafers through a Silicon-on-Glass (SOG) fabrication process. The fully symmetric structure of this MEMS ring gyroscope provides several advantages, including higher accuracy, enhanced shock resistance, broader dynamic range, and simplified structural design, facilitating mass production.

**Figure 2:**
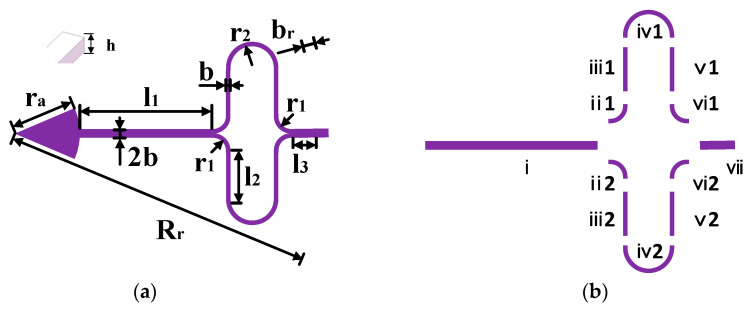
Gyroscope structural parameters: (**a**) main structural parameters of the MEMS ring gyroscope; (**b**) segmented U-shaped support beams.

The MEMS ring gyroscope consists of a fully symmetrical ring resonator supported by a central anchor, silicon electrodes, and a glass substrate with patterned electrodes. The primary structural parameters of the MEMS ring gyroscope are illustrated in Figure 2. The resonant ring structure is supported by a central anchor, connected to silicon electrodes and a patterned glass substrate. The symmetrical design offers multiple advantages, including enhanced accuracy, improved shock resistance, a wider dynamic range, and simplified structure facilitating mass production. The support beams consist of combinations of linear and curved segments, divided into seven sections defined sequentially along the beam path. As shown in Figure 2b, segment i connects directly to the anchor, while segment vii connects to the resonant ring. The main structural parameters of the MEMS ring gyroscope are illustrated in Table 1.

The MEMS ring gyroscope structure is driven electrostatically, sensing angular velocity through variations in capacitance. On the inner side of the resonant ring, there are 16 electrodes, and on the outer side, there are 16 electrodes. These electrodes include drive electrodes, drive feedback electrodes (DFE), sensing electrodes (SE), and sense feedback electrodes (SFE).

**Figure 3:**
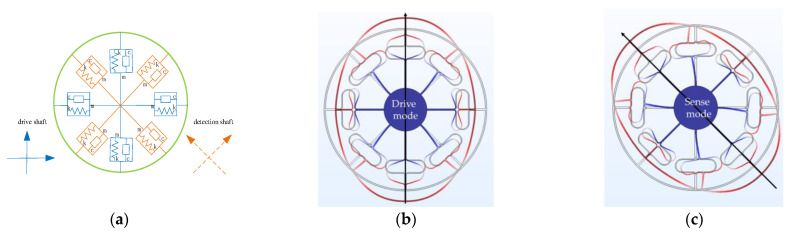
Operating principle of the MEMS ring gyroscope: (**a**) simplified second-order mechanical system model; (**b**) drive mode; (**c**) sense mode.

The vibration modes of the MEMS ring gyroscope can be categorized into the drive mode and the sense mode, each modeled as a second-order mass-spring-damper mechanical system, as depicted in Figure 3a. When no angular velocity input is present, the gyroscope operates in the drive mode. Under periodic driving forces, the ring resonator is driven to oscillate with a fixed frequency along directions at 0°, 90°, 180°, and 270°, as illustrated in Figure 3b. When angular velocity is input along the *Z*-axis perpendicular to the direction of forced vibration, the sense mode of the gyroscope is excited due to the Coriolis force. Consequently, the ring resonator generates forced bending vibrations along the directions at 45°, 135°, 225°, and 315°, as illustrated in Figure 3c.

**Figure 4:**
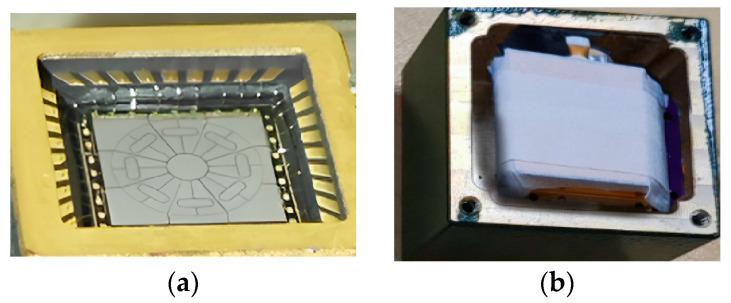
Gyroscope physical photograph: (**a**) gyroscope; (**b**) packaged prototype.

Figure 4 shows a photograph of the fabricated sensitive structure of the MEMS ring gyroscope.

## 3. Parallel Processing Methods

### 3.1. Empirical Mode Decomposition (EMD)

EMD is a data-driven adaptive signal processing method used for analyzing nonlinear and non-stationary signals. EMD adaptively decomposes a signal into a series of intrinsic mode functions (IMFs) based on its local characteristics in the time scale [26]. These oscillatory components represent different frequency bands within the signal. One of the key advantages of EMD is that it does not rely on predefined basis functions but instead performs decomposition based on the intrinsic properties of the signal. This makes it particularly suitable for processing complex signals, such as those from MEMS gyroscopes [27].

The decomposition process of empirical mode decomposition (EMD) begins by identifying all extrema (both maxima and minima) in the original data sequence *x*(*t*). Using cubic spline interpolation, an upper envelope and a lower envelope are constructed, where the lower envelope is formed by connecting all minimum points, and the upper envelope is formed by connecting all maximum points. The mean of these two envelopes, denoted as *m*_1_(*t*), is then computed. By subtracting this mean envelope *m*_1_(*t*) from the original data sequence *x*(*t*), a new data sequence *h*_1_(*t*) is obtained, as follows:

(1) $$x(t)-m_{1}(t)=h_{1}(t)$$

If the obtained *h*_1_(*t*) is not a monotonic trend, the iteration process continues. In the next iteration, *h*_1_(*t*) serves as the new original data,

(2) $$h_{1}(t)-m_{11}(t)=h_{11}(t)$$

In the equation, *m*_11_(*t*) represents the mean of the upper and lower envelopes. The smoothing process is repeated *k* times until *h*_1*k*_(*t*) becomes an intrinsic mode function (IMF), leading to the following:

(3) $$h_{1(k-1)}(t)-m_{1k}(t)=h_{1k}(t)$$

*h*_1*k*_(*t*) is the first-order intrinsic mode function (IMF) component, denoted as *a*_1_. The residual term *r*_1_ is obtained by separating *a_1_* from the original sequence, expressed as follows:

(4) $$r_{1}=x(t)-a_{1},$$

Subsequently, the residual term *r_1_* is treated as the new data for further smoothing, following the same method. This process yields a new residual term, *r*_2_ = *r*_1_ − *a*_1_. The iterative procedure continues until *r_n_ = r_n_*_−1_ − *a_n_* becomes a monotonic trend, the residual falls below a predefined threshold, or at most one extremum remains. At this point, the iteration stops. Finally, the original signal *x*(*t*) can be reconstructed from the *n*-order IMF components and the residual *r_n_*, expressed as follows:

(5) $$x(t)=\sum_{i=1}^{n}a_{i}+r_{n}$$

### 3.2. Time-Varying Filter-Based Empirical Mode Decomposition (TVFEMD)

EMD decomposes the original non-stationary and nonlinear sequence into multiple IMFs at different frequency scales. However, its limitations in mode separation and intermittency affect the ability to distinguish similar frequency components. Additionally, EMD is susceptible to noise interference, leading to mode mixing [19]. In contrast, TVFEMD introduces a significant improvement by utilizing a B-spline approximation filter for the sifting process. This approach not only preserves the advantages of conventional EMD but also effectively mitigates mode mixing and reduces sensitivity to noise [28]. Therefore, this study employs TVFEMD to minimize the non-stationarity of the original gyroscope output signal.

The sifting process of TVFEMD consists of the following steps:

Step 1: Perform the Hilbert transform on the input signal *x*(*t*), denoted as $\tilde{x}(t)$, and compute its instantaneous amplitude *A*(*t*) and instantaneous phase *ϕ*(*t*).

(6) $$A(t)=\sqrt{x{\left(t\right)}^{2}+\tilde{x}{\left(t\right)}^{2}},\;\phi (t)=\arctan[\tilde{x}(t)/x(t)]$$

The corresponding analytic signal *h*(*t*) is given as follows:

(7) $$h(t)=x(t)+i\tilde{x}(t)=A(t)e^{i\phi (t)}$$

Step 2: Identify the local maximum sequence {*t_max_*} and the local minimum sequence {*t_min_*} of the instantaneous amplitude *A*(*t*). For a multi-component signal, its analytic signal *h(t)* can also be expressed as the sum of two signal components, given as follows:

(8) $$h(t)=A(t)e^{i\phi (t)}=a_{1}(t)e^{i\phi_{1}(t)}+a_{2}(t)e^{i\phi_{2}(t)},$$

In the equation, *a_i_*(*t*) represents the amplitude of the *i*-th component, and *ϕ_i_*(*t*) denotes the phase of the *i*-th component. Thus, we obtain the following:

(9) $$A^{2}(t)=a_{1}^{2}(t)+a_{2}^{2}(t)+2a_{1}(t)a_{2}(t)\cos[\phi_{1}(t)-\phi_{2}(t)],\;\begin{array}{ll} \phi^{\prime }(t) & =\frac{1}{A^{2}(t)}\left\{\phi_{1^{\prime }}(t)\left[a_{1}^{2}(t)+a_{1}(t)a_{2}(t)\cos[\phi_{1}(t)-\phi_{2}(t)]\right]+\phi_{2^{\prime }}(t)\left[a_{2}^{2}(t)+a_{1}(t)a_{2}(t)\cos[\phi_{1}(t)-\phi_{2}(t)]\right]\right\}\\ & +\frac{1}{A^{2}(t)}\left\{a_{1^{\prime }}(t)a_{2}(t)\sin[\phi_{1}(t)-\phi_{2}(t)]-a_{1}(t)a_{2^{\prime }}(t)\sin[\phi_{1}(t)-\phi_{2}(t)]\right\} \end{array}$$

Assuming that *A*(*t*) attains a local minimum at *t_min_*, we can express the following:

(10) $$\cos[\phi_{1}(t_{\min})-\phi_{2}(t_{\min})]=-1,$$

Substituting the given expressions, we obtain the following:

(11) $$A(t_{\min})=|a_{1}(t_{\min})-a_{2}(t_{\min})|,\;\phi^{\prime }(t_{\min})A^{2}(t_{\min})=\phi_{1^{\prime }}(t_{\min})[a_{1}^{2}(t_{\min})-a_{1}(t_{\min})a_{2}(t_{\min})]+\phi_{2^{\prime }}(t_{\min})[a_{2}^{2}(t_{\min})-a_{1}(t_{\min})a_{2}(t_{\min})]$$

At the same time, based on derivative operations, we obtain the following:

(12) $$a_{1}^{\prime }(t_{\min})-a_{2}^{\prime }(t_{\min})=0,$$

Using the four equations above, we can calculate *a*_1_(*t_min_*), *a*_2_(*t_min_*), *ϕ*_1_*′*(*t_min_*) and *ϕ*_2_*′*(*t_min_*). Similarly, by solving the same set of equations, we can determine *a*_1_*′*(*t_max_*), *a*_2_*′*(*t_max_*), *ϕ*_1_*′*(*t_max_*), and *ϕ*_2_*′*(*t_max_*).

(13) $$\cos[\phi_{1}(t_{\max})-\phi_{2}(t_{\max})]=1,\;A(t_{\max})=a_{1}(t_{\max})+a_{2}(t_{\max}),\;\phi^{\prime }(t_{\max})A^{2}(t_{\max})=\phi_{1}^{\prime }(t_{\max})[a_{1}^{2}(t_{\max})+a_{1}(t_{\max})a_{2}(t_{\max})]+\phi_{2}^{\prime }(t_{\max})[a_{2}^{2}(t_{\max})+a_{1}(t_{\max})a_{2}(t_{\max})],\;{a_{1}}^{\prime }(t_{\max})+{a_{2}}^{\prime }(t_{\max})=0$$

Step 3: Compute the amplitudes *a*_1_(*t*) and *a*_2_(*t*). Taking the following,

(14) $$r_{1}(t)=\left|a_{1}(t)-a_{2}(t)\right|,\;r_{2}(t)=a_{1}(t)+a_{2}(t)$$

We obtain the following:

(15) $$r_{1}(t_{\min})=A(t_{\min})=|a_{1}(t_{\min})-a_{2}(t_{\min})|,\;r_{2}(t_{\max})=A(t_{\max})=a_{1}(t_{\max})+a_{2}(t_{\max})$$

Since *a*_1_(*t*) and *a*_2_(*t*) vary slowly, *r*_1_(*t*) and *r*_2_(*t*) can be estimated through the interpolation of *A*({*t_min_*}) and *A*({*t_max_*}), respectively. Thus, *a*_1_(*t*) and *a*_2_(*t*) can be computed by solving the corresponding equations.

(16) $$a_{1}(t)=\left[r_{1}(t)+r_{2}(t)\right]/2,\;a_{2}(t)=\left[r_{2}(t)-r_{1}(t)\right]/2$$

Step 4: Compute *ϕ*_1_*′*(*t*) and *ϕ*_2_*′*(*t*). Taking the following:

(17) $$\gamma_{1}(t)={\phi_{1}}^{\prime }(t)[a_{1}^{2}(t)-a_{1}(t)a_{2}(t)]+{\phi_{2}}^{\prime }(t)[a_{2}^{2}(t)-a_{1}(t)a_{2}(t)],\;\gamma_{2}(t)={\phi_{1}}^{\prime }(t)[a_{1}^{2}(t)+a_{1}(t)a_{2}(t)]+{\phi_{2}}^{\prime }(t)[a_{2}^{2}(t)+a_{1}(t)a_{2}(t)]$$

We obtain the following:

(18) $$\gamma_{1}(t_{\min})=\phi^{\prime }(t_{\min})A^{2}(t_{\min})=\phi_{1}^{\prime }(t_{\min})[a_{1}^{2}(t_{\min})-a_{1}(t_{\min})a_{2}(t_{\min})]+\phi_{2}^{\prime }(t_{\min})[a_{2}^{2}(t_{\min})-a_{1}(t_{\min})a_{2}(t_{\min})],\;\gamma_{2}(t_{\max})=\phi^{\prime }(t_{\max})A^{2}(t_{\max})=\phi_{1}^{\prime }(t_{\max})[a_{1}^{2}(t_{\max})+a_{1}(t_{\max})a_{2}(t_{\max})]+\phi_{2}^{\prime }(t_{\max})[a_{2}^{2}(t_{\max})+a_{1}(t_{\max})a_{2}(t_{\max})]$$

Thus, *ϕ*_1_*′*(*t*) and *ϕ*_2_*′*(*t*) can be expressed as follows:

(19) $$\phi_{1}^{\prime }(t)=\frac{\gamma_{1}(t)}{2a_{1}^{2}(t)-2a_{1}(t)a_{2}(t)}+\frac{\gamma_{2}(t)}{2a_{1}^{2}(t)+2a_{1}(t)a_{2}(t)},\;\phi_{2}^{\prime }(t)=\frac{\gamma_{1}(t)}{2a_{2}^{2}(t)-2a_{1}(t)a_{2}(t)}+\frac{\gamma_{2}(t)}{2a_{2}^{2}(t)+2a_{1}(t)a_{2}(t)}$$

Step 5: Compute the local cutoff frequency using the following formula:

(20) $$\phi_{\mathrm{bis}}^{\prime }(t)=\frac{\phi_{1}^{\prime }(t)+\phi_{2}^{\prime }(t)}{2}=\frac{\gamma_{2}(t)-\gamma_{1}(t)}{4a_{1}(t)a_{2}(t)}$$

Step 6: After obtaining the local cutoff frequency $\phi_{\mathrm{bis}}(t)$, the signal *g*(*t*) can be determined.

(21) $$g(t)=\cos\left[{\int \phi_{\mathrm{bis}}}^{\prime }(t)dt\right]$$

Then, the local extrema points of *g*(*t*) are used as nodes to construct a B-spline approximation filter. By applying this B-spline approximation filter to the gyroscope output sequence *x*(*t*), the filtered result *c^i^*(*t*) is obtained.

Step 7: This method defines a stopping criterion, given as follows:

(22) $$\theta (t)=\frac{B_{L}(t)}{\phi_{\mathrm{avg}}(t)}$$

If *θ*(*t*) ≤ *ζ*, then *x*(*t*) is considered an intrinsic mode function (IMF) component. Otherwise, *x*(*t*) − *c^i^*(*t*) is used as the new input signal, and Steps 1 to 6 are repeated until the stopping criterion is satisfied. In the equation, *ζ* represents the bandwidth threshold, typically set to 0.1, $\phi_{\mathrm{avg}}(t)$ denotes the weighted mean instantaneous frequency, and *B_L_*(*t*) is the instantaneous bandwidth.

Finally, after the TVFEMD decomposition, the original input signal *x*(*t*) is decomposed into S sub-sequences {*c^i^*(*t*)∣*i =* 1, 2, …, *S*}, satisfying the following condition:

(23) $$x(t)=\sum_{i=1}^{S}c^{i}(t)$$

where *c^i^*(*t*) represents the *i*-th sub-sequence.

### 3.3. Particle Swarm Optimization (PSO)

When TVFEMD is utilized to realize adaptive decomposition of signals, its performance is highly dependent on the setting of the bandwidth threshold (bandwidth control parameter) and B-spline order (filter smoothness parameter). In traditional applications, parameter selection mostly relies on empirical trial-and-error methods or fixed empirical values but suffers from the defects of high subjectivity and insufficient adaptability. This section proposes an adaptive configuration method for TVFEMD parameters using the particle swarm optimization algorithm (PSO).

PSO is a global optimization algorithm [29]. The population consists of M particles, represented as *X =* (*X*_1_, *X*_2_, …, *X_m_*). Each particle’s position is described by a D-dimensional vector *X_i_ =* (*X_i_*_1_, *X_i_*_2_,…, *X_iD_*), while its velocity is given by *V_i_ =* (*v_i_*_1_, *v_i_*_2_,…, *v_iD_*). The local best position of an individual particle is denoted as *Q_i_ =* (*q_i_*_1_, *q_i_*_2_,…,*q_iD_*), and the global best position of the entire swarm is represented as *G =* (*g_i_*_1_, *g_i_*_2_,…,*g_D_*). Each particle updates its position and velocity according to the following equations:

(24) $$\nu_{id}^{a+1}=\omega \nu_{id}^{a}+c_{1}\eta (q_{id}^{a}-x_{id}^{a})+c_{2}\eta (g_{d}^{a}-x_{id}^{a}),\;x_{id}^{a+1}=x_{id}^{a}+v_{id}^{a+1}$$

where *w* is the inertia weight; *d* = 1, 2, …, *D* represents the dimension index; *i* = 1, 2, …, *M* denotes the particle index; *a* is the current iteration number; *c*_1_ and *c*_2_ are the acceleration coefficients; η is a random number uniformly distributed in [0, 1].

### 3.4. Sample Entropy (SE)

Sample entropy (SE) is a probability-based statistical method used to effectively quantify the complexity of a time series. Its fundamental principle is to measure the probability of new pattern generation within the sequence—higher entropy values indicate greater complexity in the time series [30]. The computation steps of sample entropy are as follows:

Step 1: Define the time series length as *L*, and construct a set of *m*-dimensional subsequences in chronological order, expressed as follows:

(25) U(i)={u(i),u(i+1),…,u(i+m−1)},    i=1,2,…,L−m+1

Step 2: Compute the distance between vectors using the following formula:

(26) $$d_{ij}=\max_{a=0,\ldots ,m-1}|U(i+a)-U(j+a)|$$

Step 3: Determine the similarity threshold and count the number of matching vectors. Set the similarity threshold *r*, then count the number of vectors satisfying *d_ij_* ≤ *r* and define it as *B_i_^m^*(*r*):

(27) $$B_{i}^{m}(r)=\frac{B_{i}}{L-m+1}$$

Step 4: Compute the sample entropy (SE) by extending the dimension to *m* + 1 and calculating the matching probability:

(28) $$B^{m}(r)=\frac{1}{L-m+1}\sum_{i=1}^{L-m+1}B_{i}^{m}(r)$$

Finally, based on the definition of information entropy, the formula for sample entropy (SE) is given as follows:

(29) $$SampEn(m,r,L)=-\ln\frac{B^{m+1}(r)}{B^{m}(r)}$$

When the sample entropy (SE) value is low, it indicates that the signal sequence has high similarity and strong regularity, making it a valid signal. Conversely, if the SE value is high, it suggests that the sequence is more disordered with weaker regularity, potentially consisting mainly of noise.

### 3.5. Time–Frequency Peak Filtering (TFPF)

In the temperature characteristic components obtained through TVFEMD decomposition, the low-frequency noise segments typically exhibit broadband random disturbances, while the mixed segments contain a complex coupling of temperature drift trends and high-frequency noise. Traditional global filtering methods often lead to feature waveform distortion or residual noise retention when dealing with such non-uniform noise.

Time–frequency peak filtering (TFPF) addresses this issue by mapping the signal’s time–frequency distribution ridge to an instantaneous frequency estimate, enabling noise energy redistribution in the joint time–frequency domain. This method is particularly effective for suppressing non-Gaussian and non-stationary noise in MEMS gyroscope temperature signals [31]. This method is primarily based on the Wigner–Ville distribution (WVD). By applying filtering and noise reduction, it effectively enhances signal quality.

The TFPF approach modulates the noisy signal into an analytic signal and computes its pseudo-Wigner–Ville distribution (PWVD). The peak values of the PWVD spectrum are then used as instantaneous frequency estimates, enabling an unbiased time-domain signal estimation to suppress noise.

Assume that the noisy signal *y*(*t*) is given as follows:

(30) y(t)=x(t)+n(t)

where *x*(*t*) represents the useful signal, and *n*(*t*) denotes the noise. The specific steps for denoising *y*(*t*) using Time–Frequency Peak Filtering (TFPF) are as follows:

Step 1: Perform modulation analysis on the noisy signal. The analytic signal *z*(*t*) is given as follows:

(31) $$z(t)=e^{j2\pi \mu \int_{0}^{t}y(\lambda )d\lambda }$$

where *μ* is the frequency modulation factor, with a value range of [0, 1].

Step 2: Compute the pseudo-Wigner–Ville distribution (PWVD) of the analytic signal *z*(*t*) as follows:

(32) $$PW_{2}(t,f)=\int_{-\infty }^{\infty }h(\tau )z\left(t+\frac{\tau }{2}\right)z^{*}\left(t-\frac{\tau }{2}\right)e^{-j2\pi ft}d\tau$$

where *t* denotes time, *τ* serves as the integration variable, *f* represents frequency, *z*^∗^ is the complex conjugate of *z*, and *h*(*τ*) functions as the window function.

Step 3: Search for the peak values in the time–frequency distribution as the estimated instantaneous frequency, obtaining the estimated useful signal as follows:

(33) $$f_{z}(t)=\frac{\mathrm{argmax}[PW_{z}(t,f)]}{\mu }$$

The window length of TFPF significantly impacts its denoising performance. A long window enhances noise suppression but may introduce signal distortion, whereas a short window retains signal integrity but provides relatively weaker denoising capability.

### 3.6. Long Short-Term Memory Neural Network (LSTM)

The long short-term memory (LSTM) neural network is highly effective in analyzing time-series data. In time-series problems, making appropriate use of current and past input data to predict the data at t + k enables better extraction and utilization of input information. Therefore, the LSTM neural network is chosen for handling gyroscope temperature drift bias data [32].

**Figure 5:**
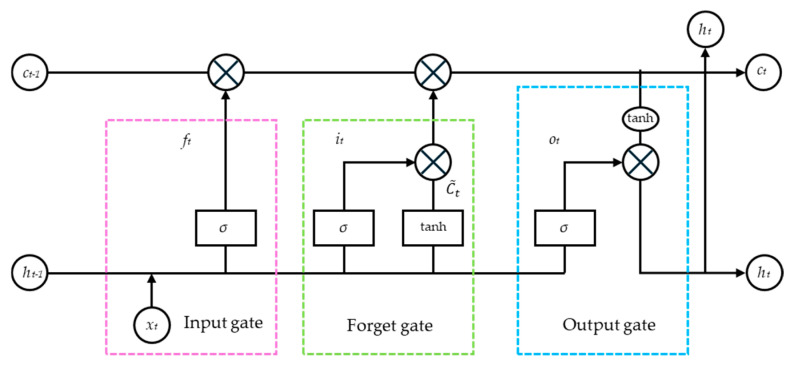
Schematic diagram of the LSTM network structure.

The LSTM network builds upon the recurrent neural network (RNN) by incorporating three gating units: the forget gate, input gate, and output gate. Its basic structure is illustrated in Figure 5. The network primarily relies on these three gating mechanisms to selectively retain and update information, thereby enhancing its capability to model time-series data. Specifically, the forget gate determines how much of the previous state information should be discarded, based on the previous cell output and the new input data. The input gate assigns weights to new information and integrates it into the previous state matrix. Simultaneously, it updates the new memory cell state based on the previous output value and the current input value. By combining the operations of the forget gate and input gate, the network generates an updated state representation.

Forget gate internal calculation formula:

(34) $$f_{t}=\sigma (W_{f}\cdot [h_{t-1},x_{t}]+b_{f})$$

Input gate internal calculation formula:

(35) $$\left\{\begin{array}{l} i_{i}=\sigma (W_{i}\cdot [h_{i-1},x_{t}]+b_{i})\\ {\tilde{C}}_{t}=tanh(W_{C}\cdot [h_{t-1},x_{t}]+b_{C})\\ C_{t}=f_{t}\cdot C_{t-1}+i_{t}\cdot {\bar{C}}_{t} \end{array}\right.$$

Output gate internal calculation formula:

(36) $$\left\{\begin{array}{l} o_{t}=\sigma (W_{o}[h_{t-1},x_{t}]+b_{o})\\ h_{t}=o_{t}\cdot tanh(C_{t}) \end{array}\right.$$

Before using the LSTM network, it is essential to determine its structure and parameters, as improper configuration may degrade LSTM performance, ultimately affecting temperature compensation accuracy. Key parameters include the number of hidden layer neurons, block size, maximum training epochs, and learning rate.

### 3.7. Football Team Training Algorithm (FTTA)

The football team training algorithm (FTTA) simulates the player position optimization and tactical collaboration mechanism and shows strong global optimization ability and convergence stability in solving high-dimensional parameter space optimization problems by constructing a three-phase search strategy of “attack-defense-balance”. The FTTA dynamically adjusts the hyperparameters of the LSTM network, which breaks through the bottleneck of the optimization efficiency of traditional heuristic algorithms. The algorithm simulates three stages of the training session: collective training, group training, and individual extra training.

#### 3.7.1. Team Training

At the start of the training process, players participate in team training under the supervision of a coach. They are classified into four distinct roles: followers, discoverers, thinkers, and volatilities. In each iteration, player roles are reassigned randomly.

Followers: Followers adjust their position based on the location of the best player to maintain a relative position with them. The update formula is given by the following:

(37) $$F_{i,j}^{k}\mathrm{new}=F_{i,j}^{k}\mathrm{old}+\mathrm{rand}\times (\begin{array}{c} F_{Best,j}^{k}-F_{i,j}^{k}\mathrm{old}) \end{array}$$

Discoverers: Discoverers explore positions between the best and worst players to identify potential new locations. The update formula is given by the following:

(38) $$F_{i,j}^{k}\mathrm{new}=F_{i,j}^{k}\mathrm{old}+{\mathrm{rand}}_{1}\times (F_{Best,j}^{k}-F_{i,j}^{k}\mathrm{old})-{\mathrm{rand}}_{2}\times (F_{Worst,j}^{k}-F_{i,j}^{k}\mathrm{old})$$

Thinkers: Thinkers adjust their position based on the best player’s location, but with a smaller adjustment magnitude. The update formula is given by the following:

(39) $$F_{i,j}^{k}\mathrm{new}=F_{i,j}^{k}\mathrm{old}+\mathrm{rand}*(F_{Best,j}^{k}-F_{Worst,j}^{k})$$

Volatilities: Volatilities adjust their position based on their current state, simulating a self-enhancement process. The update formula is given by the following:

(40) $$F_{i,j}^{k}\mathrm{new}=F_{i,j}^{k}\mathrm{old}\times (1+t(k))$$

#### 3.7.2. Group Training

After completing team training, players are categorized into four specific roles based on their characteristics: forwards, midfielders, defenders, and goalkeepers. Each group undergoes specialized training to enhance its respective skill levels. During this phase, coordination and teamwork among players are significantly strengthened.

#### 3.7.3. Individual Extra Training

After group training, players undergo individual extra training based on their personal needs. The update formula is given by the following:

(41) $$F_{\mathrm{Best}}^{k,\mathrm{new}}=F_{\mathrm{Best}}^{k,\mathrm{old}}\cdot \left(1+\left(1-\frac{1}{k}\right)\cdot N+\frac{1}{k}\cdot C\right)$$

*N* denotes the Gaussian distribution, and *C* denotes the Cauchy distribution. The two are used to control the algorithm’s local development and global exploration capabilities, respectively. *C* and *N* joint variation is used to describe individual extra training, and *k* represents the number of iterations. The reason for choosing the Gaussian–Cauchy distribution is that, during the early stages of training, most players are generally not at a high level. Therefore, the best player has a higher probability of receiving a significant boost. At this stage, the Cauchy distribution dominates, allowing for a broader range of improvements for players, which is beneficial for the global search. As the number of iterations increases, improving players’ abilities becomes more challenging. At this point, the Gaussian distribution becomes more influential, and the range of improvement for the players gradually narrows, which is more conducive to local search.

By mimicking the training dynamics of a football team, FTTA significantly improves the algorithm’s convergence efficiency, helping to avoid local optima and ultimately leading to the global optimal solution.

### 3.8. Temperature Compensation Model

**Figure 6:**
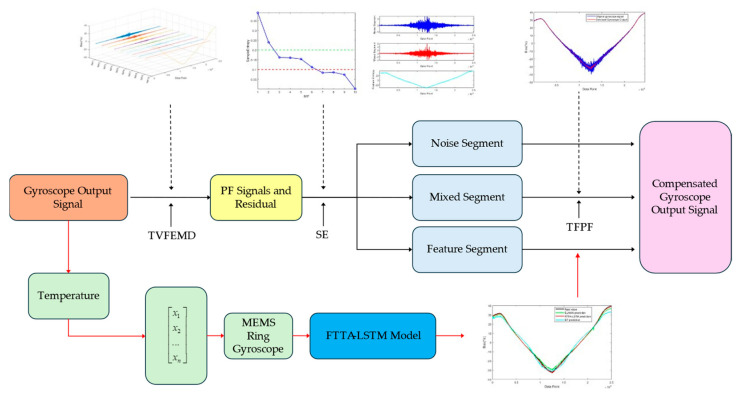
Framework of the fusion algorithm based on PSO-TVFEMD-SE-TFPF and FTTA-LSTM models.

Based on the above algorithms, this paper proposes a parallel denoising and temperature compensation fusion algorithm for MEMS ring gyroscopes, integrating PSO-TVFEMD-SE-TFPF and FTTA-LSTM models. The architecture of the algorithm is illustrated in Figure 6, and the specific steps are as follows.

#### 3.8.1. Optimizing TVFEMD Decomposition Parameters Using PSO

PSO is applied to optimize the decomposition parameters of TVFEMD, ensuring the best decomposition performance. Next, sample entropy (SE) is employed to evaluate each component concerning continuity, autocorrelation, and complexity, categorizing the signal into three sections: noise, mixed, and feature segments. The noise segment demonstrates chaotic behavior akin to white noise, whereas the mixed segment consists of both noise and drift trends. To process the mixed segment, the time–frequency peak filtering (TFPF) algorithm is employed.

#### 3.8.2. Temperature Compensation Using the FTTA-LSTM Model

To improve the accuracy of the algorithm, the FTTA-LSTM model is used for temperature compensation. This model optimizes prediction error and weight range as objective functions. FTTA is applied for multi-objective optimization of the LSTM neural network parameters. The optimized FTTA-LSTM model is then used to compensate for the feature segment, providing a more accurate prediction of temperature drift at the current moment while better correlating with its impact on MEMS gyroscope output. After training, the model delivers an accurate prediction of temperature drift at the current moment.

#### 3.8.3. Generating the Final Compensation Signal

By reconstructing the processed gyroscope output, the final compensated signal is derived. This outcome effectively mitigates temperature-induced errors, successfully removing both high-frequency noise and low-frequency drift.

## 4. Experiment

### 4.1. The Temperature Experimental Process

**Figure 7:**
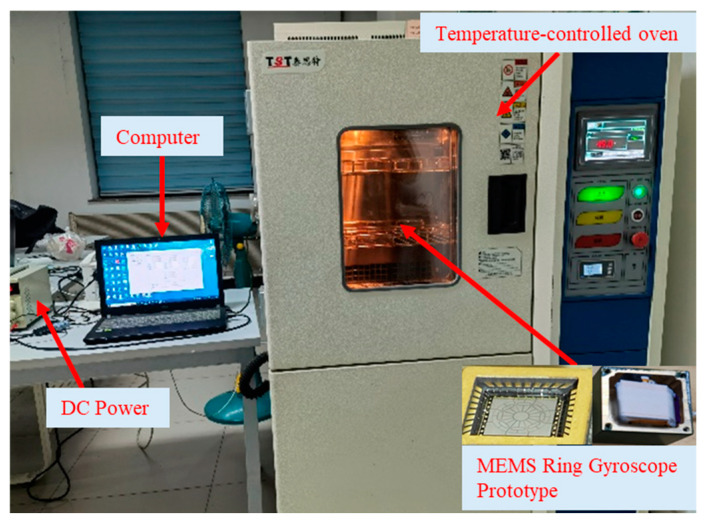
Full-temperature testing equipment for the MEMS ring gyroscope.

To validate the proposed parallel model, a temperature drift experiment was conducted. The experimental setup is illustrated in Figure 7. Inside the temperature chamber, a packaged MEMS ring gyroscope, an integrated circuit (ASIC), and a data acquisition unit were placed. The ASIC and data acquisition unit, provided by Beijing Shuimu Zhixin Technology Co., Ltd. (Beijing, China), were powered by a +7 V DC regulated power supply. The collected data were transmitted to the host computer via an RS232-to-USB serial connection.

The temperature experiment was carried out following IEEE Standard 1431 [33] and IEEE Standard 1554 [34]. The temperature range was controlled between −40 °C and 60 °C. Within this range, the system underwent a continuous heating and cooling process at 0.5 °C intervals. At each temperature point, the gyroscope’s bias signal was measured and recorded for 60 min to ensure an accurate assessment of temperature drift characteristics.

Step 1: The temperature chamber was first set to 300 K (room temperature). After powering on the gyroscope, it was kept at this temperature for 30 min to allow it to reach a stable operating condition before initiating the temperature experiment.

Step 2: The temperature was raised to 60 °C, allowing sufficient time for the gyroscope’s internal temperature to equilibrate with the chamber environment. To ensure stability, the gyroscope remained at 60 °C for 30 min before proceeding with further measurements.

Step 3: Multiple sets of temperature experiments were conducted to ensure data consistency.

### 4.2. The Experimental Results

**Figure 8:**
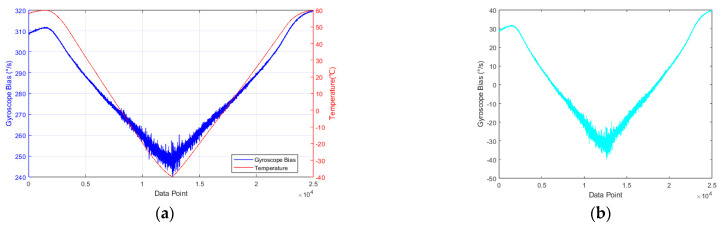
Full-temperature test data of MEMS ring gyroscope: (**a**) collected temperature and gyroscope zero-bias output; (**b**) gyroscope zero-bias after mean subtraction.

The zero-bias output data of the MEMS ring gyroscope and its temperature variations are shown in Figure 8a. From the figure, it is evident that as the temperature changes, the gyroscope output signal becomes more pronounced, exhibiting a nonlinear relationship. When the temperature decreases from 60 °C to −40 °C, the gyroscope bias shifts from 311.8°/s to 241.1°/s. Furthermore, in addition to the temperature drift, the gyroscope output signal contains a significant amount of noise. This observation clearly indicates that temperature has a considerable impact on the accuracy of the MEMS ring gyroscope output. Since the gyroscope has a constant bias error under stable room temperature conditions, mean subtraction is applied to the gyroscope zero-bias data, yielding the gyroscope output error influenced by both temperature and noise, as shown in Figure 8b.

**Figure 9:**
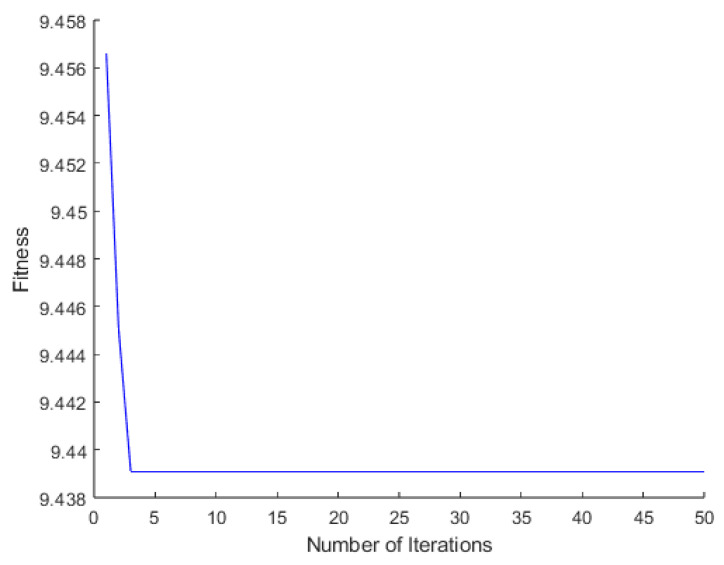
Fitness curve during the PSO iteration process.

According to the steps of the proposed temperature error processing fusion algorithm, the output signal is first decomposed using TVFEMD. Before decomposition, PSO is employed to determine the optimal parameters for TVFEMD, including the bandwidth threshold and B-spline order, denoted as [ζ, n]. The PSO parameter settings are listed in Table 2, and the convergence evolution process is illustrated in Figure 9. As a result, the optimal decomposition parameters for TVFEMD are obtained as [ζ, n] = [0.1, 20].

**Figure 10:**
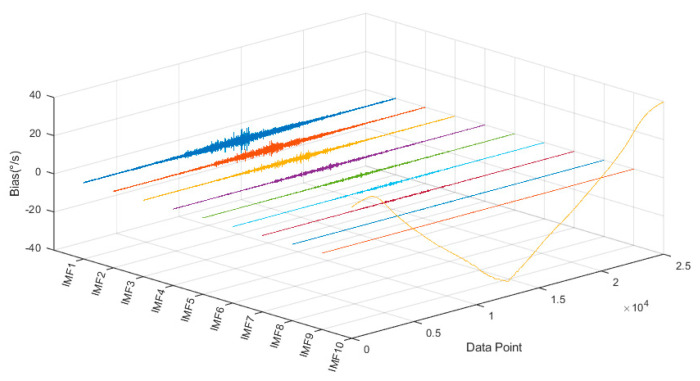
TVFEMD decomposition of the output signal.

Figure 10 illustrates the TVFEMD decomposition of the output signal. After decomposition, the adaptive method generates 10 frequency band components.

Subsequently, the SE algorithm was used to calculate the SE values of 10 PF components and the Res component. Based on their SE values, these 10 components were classified into three categories.

**Figure 11:**
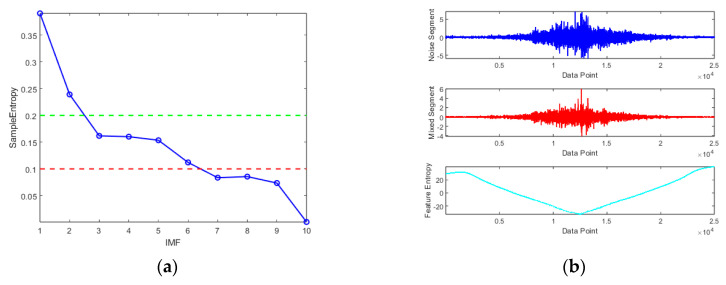
SE algorithm calculation and classification: (**a**) SE values of each IMF component; (**b**) segmentation of signal sections.

As shown in Figure 11, the SE values range from 0 to 0.5. Components with SE values greater than 0.2 (including PF1 and PF2) do not exhibit drift with temperature variations, indicating that they primarily contain noise. Therefore, this segment is identified as the noise segment and is discarded according to the fusion algorithm framework.

The signal composed of PF3 to PF6 has SE values between 0.1 and 0.2. While this segment also exhibits noise characteristics, it shows some drift as the temperature changes, categorizing it as the mixed segment. Since TFPF effectively preserves useful features during the denoising process, it is applied to this segment to remove noise while retaining essential components.

The remaining PF components and residual components with SE values below 0.075 exhibit a generally smooth curve and show significant drift with temperature variations, identifying them as the feature segment. Therefore, FTTA-LSTM is used to perform prediction and compensation for this part.

**Figure 12:**
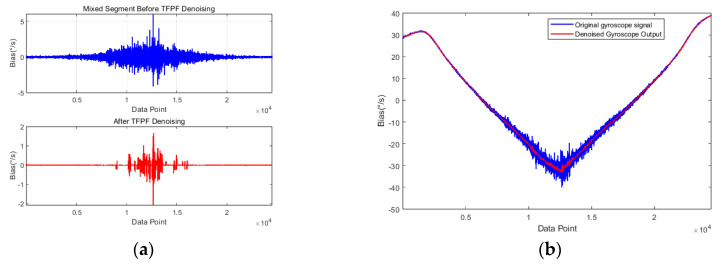
Comparison of TFPF denoising performance: (**a**) comparison of the mixed segment before and after denoising; (**b**) comparison of the gyroscope output signal before and after denoising.

To evaluate the denoising capability of the PSO-TVFEMD-SE-TFPF framework, we reconstructed the denoised mixed segment and the denoised gyroscope output signal. As shown in Figure 12, the noise in the signal is largely eliminated. To quantitatively assess the denoising performance of the PSO-TVFEMD decomposition, we compared the signals before and after denoising, as summarized in Table 3. The data clearly demonstrate a significant reduction in noise for the denoised signal, which greatly facilitates subsequent temperature compensation. These results highlight the superiority of the proposed model.

Before establishing the temperature compensation model to compensate for the feature segment, the FTTA algorithm was applied to optimize the LSTM network, ensuring the optimal network parameters.

**Figure 13:**
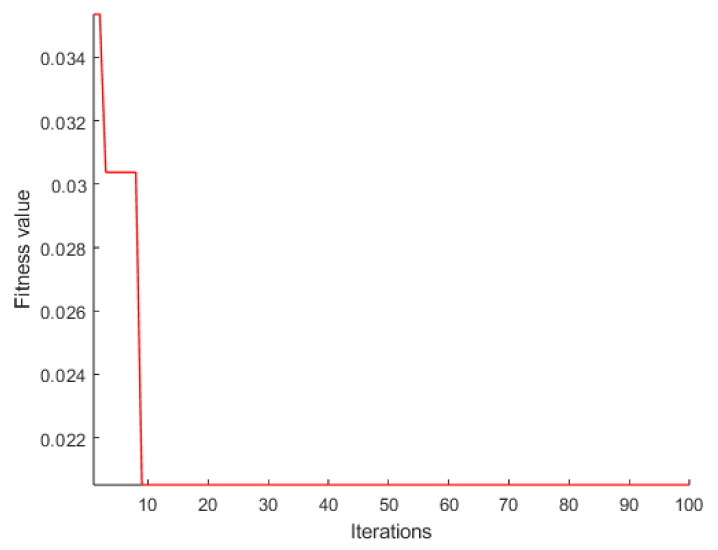
Fitness convergence of the FTTA optimization process for LSTM.

As previously mentioned, after FTTA optimization and iteration, the optimized parameters were determined as follows: number of hidden layer neurons in the LSTM network: 100; block size: 3; maximum training epochs: 1000, and learning rate: 0.01. The FTTA algorithm parameter settings are listed in Table 4, and the fitness convergence process during optimization is illustrated in Figure 13.

**Figure 14:**
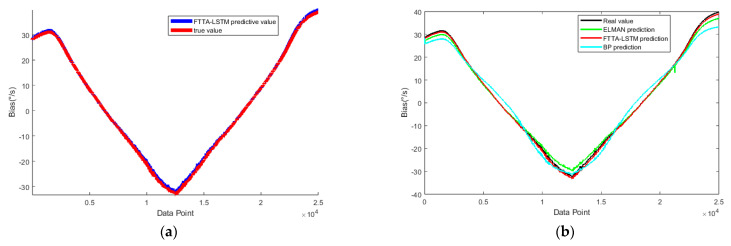
LSTM network prediction for the feature segment: (**a**) prediction using the FTTA-LSTM network; (**b**) comparison of predictions across different algorithms.

The FTTA-optimized LSTM network was used to predict the feature segment, and the results were compared with those from BP and ELMAN networks. The obtained results are shown in Figure 14.

**Figure 15:**
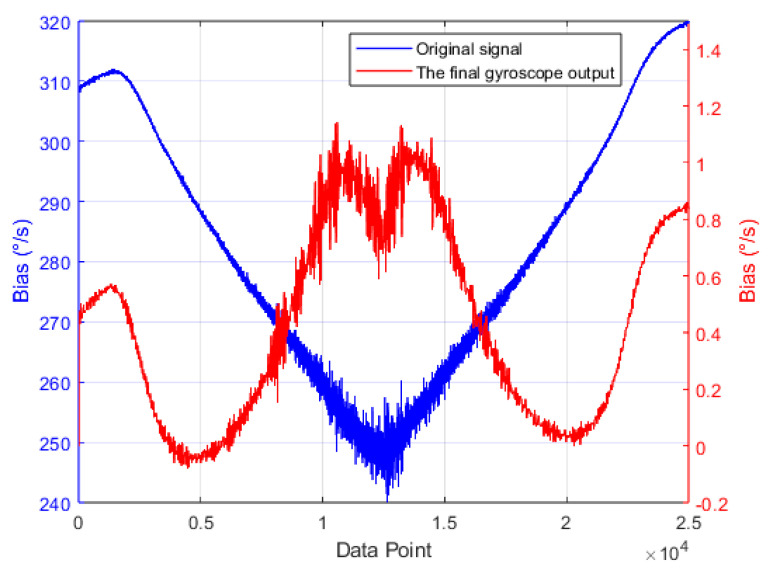
Comparison of full-temperature zero-bias output of the gyroscope before and after compensation using the fusion algorithm.

The full-temperature zero-bias output curve of the gyroscope before and after compensation is shown in Figure 15. It can be observed that after applying the proposed compensation algorithm, the temperature-induced drift is effectively eliminated.

As an error analysis method defined in the IEEE Standard [35], Allan variance is a time-domain analysis technique commonly used for random error modeling in inertial sensor data. In this study, the Allan variance curve analysis is applied to perform a quantitative evaluation of the angular random walk (ARW) and bias instability (BI) of both the raw gyroscope output and the compensated gyroscope output after applying the PSO-TVFEMD-SE-TFPF and FTTA-LSTM parallel denoising and fusion compensation algorithm.

**Figure 16:**
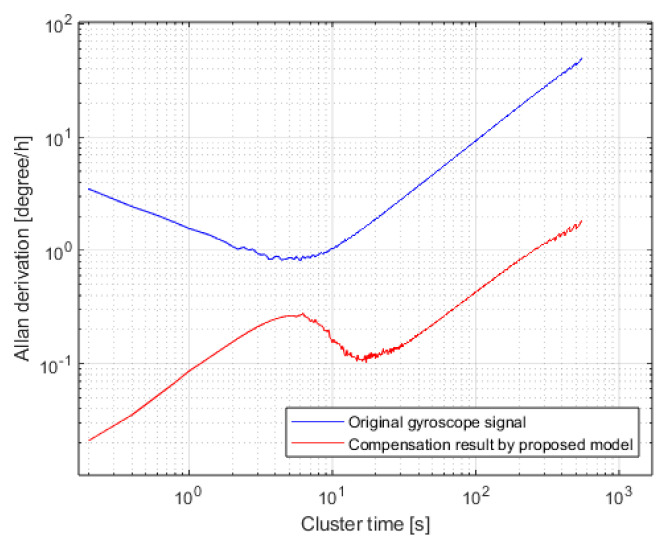
Allan variance evaluation.

The Allan variance results for both the raw gyroscope output signal and the compensated output after applying the proposed fusion algorithm are shown in Figure 16. The temperature-compensated zero-bias data demonstrates a significant improvement under varying temperature conditions. For angular random walk (ARW), the minimum value before compensation was 3.48°/√h, while after compensation, it was reduced to 0.02°/√h, achieving a 99.43% improvement in ARW performance. For bias instability (BI), the lowest value before compensation was 96.51°/h, whereas, after compensation, it was reduced to 2.23°/h, resulting in a 97.69% enhancement in BI performance.

These results confirm that within the −40 °C to 60 °C temperature range, the proposed temperature error fusion compensation algorithm effectively mitigates temperature drift and removes both low-frequency and high-frequency noise from the gyroscope output signal, thereby significantly enhancing the temperature adaptability of the MEMS ring gyroscope.

## 5. Conclusions

This study investigates the structure of MEMS ring gyroscopes and proposes a temperature compensation and denoising solution. A novel parallel fusion compensation method based on PSO-TVFEMD-SE-TFPF and FTTA-LSTM is introduced. Given that MEMS ring gyroscope signals vary under different temperature conditions and that the noise and temperature drift in the output signals differ, the PSO-optimized TVFEMD algorithm is used for signal decomposition. After decomposition, the sample entropy (SE) values of each component are calculated to assess their complexity, and the signal is classified into noise, mixed, and temperature feature segments. Different processing methods are applied to each segment to suit their respective characteristics. This fusion algorithm combines time-series modeling with LSTM and optimizes TVFEMD and LSTM using PSO and FTTA algorithms, enhancing gyroscope temperature error compensation. The FTTA-LSTM model effectively addresses traditional local optimization issues, improving the accuracy and robustness of temperature compensation. The method’s effectiveness is verified through temperature experiments, output comparisons, and Allan variance analysis, demonstrating its innovation and superiority over previous algorithms. Currently, the fusion algorithm relies on offline-optimized parameters. Future work could incorporate online learning mechanisms, such as incremental PSO, to handle rapid temperature transients.

## Figures and Tables

**Table 1:** Main structural parameters of the MEMS ring gyroscope.

Parameter	Characteristic	Parameter	Characteristic
r_a_	Radius of anchor	R_r_	Radius of resonant ring
l_1_	Length of straight beam i	b	Width of beam ii, iii, iv, v, vi
r_1_	Radius of curved beam ii, v	2b	Width of beam i, vii
l_2_	Length of straight beam iii, vi	b_r_	Width of the resonant ring
r_2_	Radius of curved beam iv	h	Thickness of the structural
l_3_	Length of straight beam vii	d	Electrode gap

**Table 2:** PSO parameter settings.

Parameter	Description
pop	Population Size
dim	Particle Dimension
[lb,ub]	Particle Position Boundaries
[vmin,vmax]	Particle Velocity Boundaries
c1 = c2	Learning Factor

**Table 3:** Output signal after denoising using PSO-TVFEMD-SE-FTTA.

Data	Mean Absolute Error (MAE)	Root Mean Square Error (RMSE)
raw data	0.4301	0.7634
PSO-TVFEMD-SE-TFPF	0.0096	0.0440

**Table 4:** FTTA Parameter settings.

Parameter	Description
pop	Population Size
dim	Particle Dimension
[lb,ub]	Particle Position Boundaries
maxgen	Number of Iterations

## Data Availability

The original contributions presented in this study are included in the article. Further inquiries can be directed to the corresponding authors.
